# Dengue Baidu Search Index data can improve the prediction of local dengue epidemic: A case study in Guangzhou, China

**DOI:** 10.1371/journal.pntd.0005354

**Published:** 2017-03-06

**Authors:** Zhihao Li, Tao Liu, Guanghu Zhu, Hualiang Lin, Yonghui Zhang, Jianfeng He, Aiping Deng, Zhiqiang Peng, Jianpeng Xiao, Shannon Rutherford, Runsheng Xie, Weilin Zeng, Xing Li, Wenjun Ma

**Affiliations:** 1 Guangdong Provincial Institute of Public Health, Guangdong Provincial Center for Disease Control and Prevention, Guangzhou, China; 2 School of Mathematics and Computing Science, Guilin University of Electronic Technology, Guilin, China; 3 Guangdong Provincial Center for Disease Control and Prevention, Guangzhou, China; 4 Centre for Environment and Population Health, Griffith University, Brisbane, Australia; Institute for Disease Modeling, UNITED STATES

## Abstract

**Background:**

Dengue fever (DF) in Guangzhou, Guangdong province in China is an important public health issue. The problem was highlighted in 2014 by a large, unprecedented outbreak. In order to respond in a more timely manner and hence better control such potential outbreaks in the future, this study develops an early warning model that integrates internet-based query data into traditional surveillance data.

**Methodology and principal findings:**

A Dengue Baidu Search Index (DBSI) was collected from the Baidu website for developing a predictive model of dengue fever in combination with meteorological and demographic factors. Generalized additive models (GAM) with or without DBSI were established. The generalized cross validation (GCV) score and deviance explained indexes, intraclass correlation coefficient (ICC) and root mean squared error (RMSE), were respectively applied to measure the fitness and the prediction capability of the models. Our results show that the DBSI with one-week lag has a positive linear relationship with the local DF occurrence, and the model with DBSI (ICC:0.94 and RMSE:59.86) has a better prediction capability than the model without DBSI (ICC:0.72 and RMSE:203.29).

**Conclusions:**

Our study suggests that a DSBI combined with traditional disease surveillance and meteorological data can improve the dengue early warning system in Guangzhou.

## Author summary

Dengue fever is an important public health problem in China, and its importance was highlighted by an unprecedented outbreak in Guangdong province in 2014. Several previous studies have found that prediction models based on internet-based data have advantages in the timely detection of dengue epidemics. In this study, we employed the Dengue Baidu Search Index (DBSI) to explore whether internet-based query data can help improve disease prediction. We found that the dengue early warning system combining DBSI with traditional surveillance and meteorological data improved the prediction capability in Guangzhou, which suggests that utilizing big data from internet search engines can provide valuable supplementary data to traditional surveillance systems particularly for developing dengue early warning systems.

## Introduction

Dengue fever (DF) is currently endemic in more than 100 countries, mainly in southeast Asia, the western Pacific islands and the Americas, with approximately 3.9 billion individuals at risk [1]. The annual number of infections is estimated at 390 million globally [2], making it one of the most significant vector-borne viral diseases.

In China, the first outbreak of DF was reported in Guangdong province in 1978 [3]. Since then DF cases have been reported in 26 provinces of China [4]. Guangdong province is the most affected areas in mainland China. In 2014, this province experienced a large outbreak resulting in 45, 224 DF cases [5,6]. Since there is no specific treatment for DF and vector control remains the most effective way to prevent and control it [2]. Early warning systems are considered as one of the prerequisites for adequate preparedness and response to DF epidemics [7]. Several previous studies have reported meteorological factors that were associated with DF outbreaks through early warning models [8–12]. Among various meteorological factors, temperature and rainfall contribute the most to dengue epidemics [13]. In Singapore, Yien et al. [12] developed a weather-based dengue-forecasting model that allows warning 16 weeks in advance of dengue epidemics with high sensitivity and specificity. However, the dengue epidemics in Guangzhou are generally characterized by low level epidemic caused by imported cases, followed by a sudden and rapid transmission [14]. They have varied greatly in size from year to year [4], which poses a different challenge for prediction than in the more stable and endemic regions. Although a study conducted by Sang *et al*. [15] attempted to develop a model based on imported cases, minimum temperature and precipitation to predict the dengue incidence in Guangzhou, DF forecasting systems still face many difficulties due to the complexity of factors influencing DF outbreaks [16].

Over the past decade the increasing number of internet users around the world has provided new sources of data potentially useful for disease surveillance. This is increasingly being recognized as an opportunity to improve traditional disease surveillance systems [17]. For example, a study reported that using Google Flu Trends (GFT) could improve the prediction of influenza trends two weeks ahead of Centers for Disease Control and Prevention (CDC) reports in the US between 2003 and 2007 [18]. Several other studies using Google, Yahoo and other search data have been conducted worldwide to predict disease trends [19–24]. However, use of GFT data is not without its problems. For example, studies found that the surveillance data did not correspond with estimates provided by the GFT model in the US during the 2009 pandemic and the 2012/2013 epidemic season [25–27]. The reasons may be related to the proportion of the population who used the internet to obtain health-related information [17], algorithm dynamics affecting Google’s search algorithm [28] and media bias [29]. Therefore, researchers believe that internet search data is a good supplement to, rather than a substitute for, traditional disease surveillance data [28].

In China, Baidu is the most popular search engine, and approximately 86.7% of internet users prefer it [30]. Some recent studies have explored the potential of using Baidu search queries to predict diseases such as influenza [31] and erythromelalgia [32]. However, there has been no similar study in utilizing such data for DF prediction in China. Therefore, the aim of this study is to examine whether an early warning model utilizing internet-based dengue query data can improve DF prediction.

## Materials and methods

### Study setting

Guangzhou, the capital city of Guangdong province, is the third most populous city in China. At the end of 2014 the population in Guangzhou was 13.1 million [33]. This city is the center of transportation, finance, industry and trade in southern China and has a large exchange in business and tourism with southeast Asia, Africa and the Indian subcontinent. It has 12 districts with an area of 7473 km^2^ and a typical subtropical monsoon climate, with an annual mean temperature of 22°C.

### Data collection

DF has been a legally notifiable communicable disease in China since 1989. Weekly DF cases in Guangzhou during the period from January 1^st^, 2011 to December 31^st^, 2014 were retrieved from China Notifiable Infectious Disease Report System (NNIDRIS). DF cases before October 2014 were diagnosed according to the China National Diagnostic Criteria for dengue fever (WS216-2008) [34], and cases after October 2014 were diagnosed according to the new version of the China National Diagnostic Criteria for dengue fever (2014 version) enacted by the National Health and Family Planning Commission (http://www.nhfpc.gov.cn).

A climate dataset was obtained from the China Meteorological Data Sharing Service System (http://cdc.nmic.cn/home.do). It included weekly average minimum temperature (°C) and cumulative rainfall (mm) from 2011–2014. The population data was collected from the Guangzhou Statistical Yearbook.

The Baidu index database (http://index.baidu.com) contains search volumes for numerous terms entered by Baidu users since January 2011. The Baidu search query data are available as daily counts at the city, province and country level. We transformed the data to weekly counts for the analysis for consistency with other time series data.

As different terms have different search volumes and can therefore produce diverse models, term selection is the critical issue in internet search data-based surveillance. However, there are no criteria in practice [32,35,36]. Previous studies generally chose the nomenclature, clinical signs and symptoms of target diseases as the main terms [23,24,32]. Related terms were obtained from a Chinese website (http://tool.chinaz.com/baidu/words.aspx). Terms suggested by the website not only include recommendations from Baidu, but also from blogs, portal websites and online reports using semantic correlation analysis [31]. Upon typing in six primary terms, we obtained a total of 32 related search terms. More terms do not necessarily lead to a better result since some recommended terms are not closely related to DF occurrence, which could reduce the detective ability of the surveillance system [32]. Hence, we filtered terms following two steps. First, we eliminated the terms irrelevant to DF and those with a search volume of zero during the study period, and after these 26 keywords remained (S1 Table). Second, Spearman’s rank correlation coefficients (ρ) were then calculated between weekly DF and search volumes. We excluded the words with correlation coefficients smaller than 0.4 (S2 Table). Weights of terms were defined by the value of the correlation coefficient. The weights calculation and Dengue Baidu Search Index (DBSI) composition formulae are as follows:

$${weight}_{i}=\frac{\rho_{i}}{\sum_{1}^{n}\rho_{i}}$$

$$Dengue\;Baidu\;Search\;Index=\sum_{1}^{n}{weight}_{i}\;{term}_{i}$$

Where *n* is the number of terms, *term*_*i*_ and *weight*_*i*_ represent the i^th^ term and the weight of it.

### Statistical methods

First, cross-correlation analysis was carried out to identify the correlation between DF occurrence with imported cases, minimum temperature, cumulative rainfall and DBSI with 1 to 16 weeks’ lag. Second, generalized additive models (GAM) were applied to fit the relationships between the variables and local DF cases. Because the variables with different time lags are highly correlated with each other, only those with maximal correlation coefficient were used to construct the model [15]. We used a cubic spline function for these variables to consider the non-linear association between factors and DF occurrence. In this study, a quasi-Poisson model was applied to allow for over-dispersion of the data. Model selection was based on the lowest generalized cross validation (GCV) scores.

In order to examine whether internet-based dengue query data could improve the prediction, we fit two models (with and without DBSI) and compared the relative parameters. Model (1) (without DBSI) and model (2) (with DBSI) are as follows:

$$Log(u_{t})=\beta_{0}+s({Tmin}_{t-e},df)+s(R_{t-b},df)+s({Imp}_{t-c},df)+s({Local}_{t-1},df)+year+s(week,df)+offset(pop)$$ (1)

$$Log(u_{t})=\beta_{0}+s(D{BSI}_{t-d},df)+s({Tmin}_{t-e},df)+s(R_{t-b},df)+s({Imp}_{t-c},df)+s({Local}_{t-1},df)+year+s(week,df)+offset(pop)$$ (2)

where *u*_*t*_ represents the predicted mean DF cases during week *t*; *s*(*Tmin*_*t–e*_,*df*) denotes the cubic spline of minimum temperature in the previous *e* weeks with corresponding df; *s*(*R*_*t–b*_,*df*) represents the cubic spline of cumulative rainfall in the previous *b* weeks with corresponding df; *s*(*Imp*_*t–c*_,*df*) represents the cubic spline of imported cases in the previous *c* weeks with corresponding df; *s*(*Local*_*t*–1_,*df*) is the autoregressive term for local DF cases in the previous week with corresponding df; *s*(*DBSI*_*t–d*_,*df*) denotes the cubic spline of DBSI in the previous *d* weeks with corresponding df; *year* is used to control long-term trend, and *s*(*week,df*) denotes the cubic spline of week with corresponding df that is used to control the seasonality; and *offset*(*pop*) accounts for population in Guangzhou during this period [12].

The df for each variable was determined according to the GCV principles and deviance explained (%) [15]. Lower GCV and higher deviance explained value indicate a better fit of the model. Finally, we chose df for *week* variable were 4, and other included variables were 3[37]; moreover, the sensitivity of the trend was tested by setting df to be 2, 3 or 4.

The disease dataset was also divided into two subsets: the first, from the 1^st^ week of 2011 to the 44^th^week of 2014 was used for model construction, and the other, from the 45^th^ to the 52^nd^ week of 2014 for external validity assessment. We used the *F* test to compare the fit of models (with or without DBSI). Moreover, intraclass correlation coefficient (ICC) and root mean squared error (RMSE) were applied to verify the consistency between the actual and predicted data [38,39].

Finally, we employed a Leave-One-Out Cross-Validation (LOOCV) method to validate the results of model (1) and model (2). LOOCV is a k-fold cross-validation method [40], and here the total dataset was divided into (n-1) subsets, where n is the number of weeks from the1^st^ week of 2011 to the 44^th^ week of 2014. In each subset, a single week’s data was removed, and the weekly number of dengue cases was predicted. Then we employed the ICC as a metric to test the correlations between predicted and observed cases.

All the analyses were performed using the “mgcv” library in R 3.2.2 [41] with a significance level of P<0.05.

## Results

**Fig 1:**
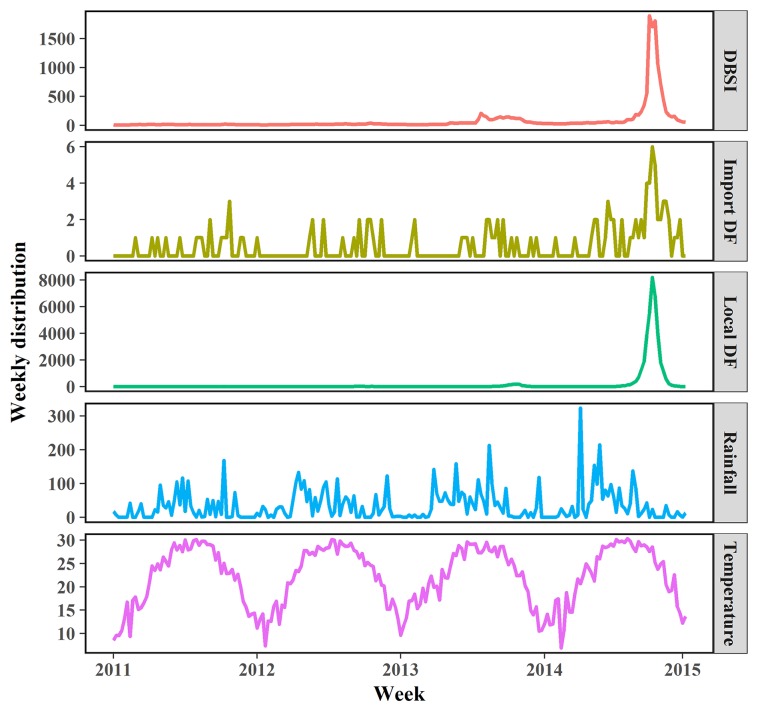
The time series of weekly rainfall, minimum temperature, DBSI, local dengue and imported dengue counts from 2011–2014 in Guangzhou.

During 2011–2014, a total of 38,860 DF cases were reported in Guangzhou city, with 116 (0.3%) imported DF cases and 38,744 (99.7%) local DF cases. A summary of meteorological variables, DBSI and DF cases are presented in Table 1. There was an average of 186.3 local DF cases and 0.6 imported DF cases every week over the study period. The mean values of the weekly DBSI, minimum temperature and cumulative rainfall were 80.8, 19.0°Cand 34.3mm, respectively. Fig 1 shows the time series of weekly meteorological variables, DBSI, and local and imported DF cases. Both a large DF outbreak and the highest weekly DBSI during the study period occurred in 2014. Weekly minimum temperature and cumulative rainfall showed an obvious seasonal pattern, peaking from June to August.

**Table 1:** Weekly meteorological parameters, DBSI, and local and imported DF cases in Guangzhou from 2011–2014.

Variables	Min	Mean	Median	Max	SD
Minimum temperature(°C)	5.4	19.0	20.3	26.6	6.3
Cumulative rainfall (mm)	0.0	34.3	16.5	324.0	46.3
DBSI	4.9	80.8	21.8	1890.3	234.8
Weekly local DF count	0.0	186.3	0.0	8200.0	929.3
Weekly imported DF count	0.0	0.6	0.0	6.0	1.0

**Fig 2:**
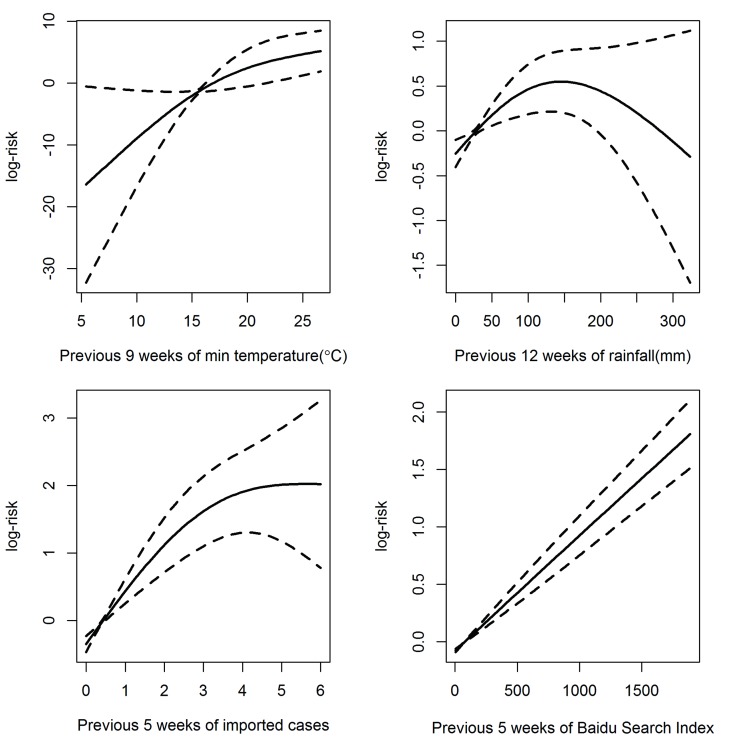
The relationship between the minimum temperature at 9 weeks’ lag, imported DF at 5 weeks’ lag, cumulative rainfall at 12 weeks lag and DBSI for the previous week with local DF cases. Note: Solid lines represent logarithmic relative risks of DF and dotted lines represent the upper and lower limits of 95% confidence intervals

The results of the cross-correlation of weekly local DF case numbers and prediction variables are shown in S3 Table. We found that minimum temperature in the previous 9 weeks, cumulative rainfall in the previous 12 weeks and imported cases in the previous 5 weeks have the highest correlation with local DF. Hence these variables were included in our model. Fig 2 shows the dose-response relationship between local DF cases and imported cases in the previous 5 weeks, minimum temperature in the previous 9 weeks, cumulative rainfall in the previous 12 weeks and DBSI in the previous week. Minimum temperature, cumulative rainfall and imported DF cases were non-linearly associated with the local DF cases. For cumulative rainfall, the risk of DF incidence increases with the increment of rainfall at first, peaking at 149mm, followed by a significant decrease. DBSI in the previous week had a positively linear relationship with the local DF.

**Fig 3:**
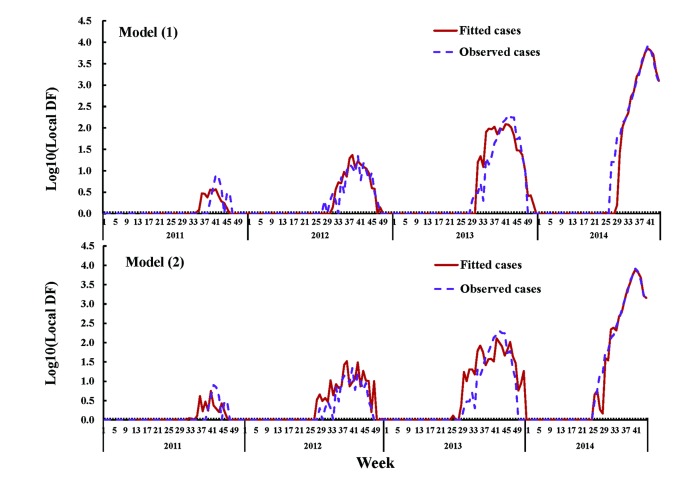
Weekly observed and fitted local DF cases using two different models from 1^st^ week of 2011 to the 44^th^ week of 2014. Note: The dotted line represents the reported dengue cases and the solid lines represent the cases fitted by the respective models.

**Fig 4:**
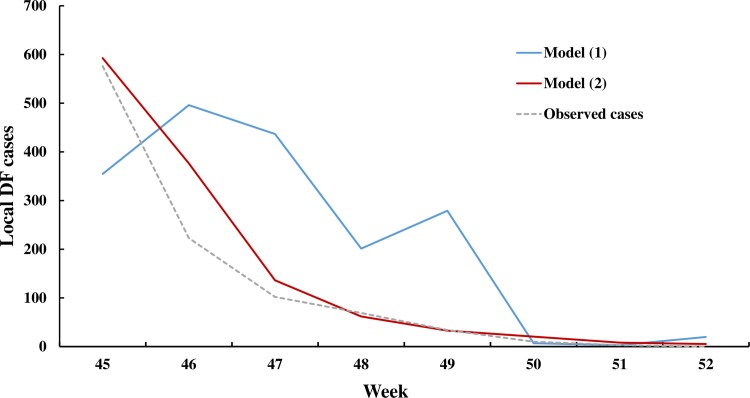
Comparison of weekly-observed DF cases from the 45^th^ week to the 52^nd^ week of 2014 with out-of-sample one-week ahead predictions. Note: The dotted line represents observed dengue cases and the solid lines show the cases predicted by the fitted models.

Fig 3 shows that both model (1) and model (2) fit the DF cases reasonably well during the training process. Our results indicate that the fit of model (1) and model (2) were both found to be significant (*F* = 10.46, *P*<0.001). The value of model with the DBSI (GCV:7.62 and Deviance explained: 99.23%) fit better than the model without DBSI (GCV: 18.41 and Deviance explained: 94.53%). Moreover, the effects of climate, imported cases and DBSI were found to be significant at the 0.05 level (S4 Table). The one-week ahead predictions of dengue outbreaks that occurred from the 45^th^ week to the 52^nd^ week of 2014 for both models are shown in Fig 4. Model (2) gives a better prediction of DF cases (ICC:0.94 and RMSE:59.86) than model (1) (ICC:0.72 and RMSE:203.29).

The results of sensitivity analyses show that the GCVs were respectively the lowest when the *df*s of weekly minimum temperature, and cumulative rainfall in model (1) and DBSI in model (2) were set to 3, which justified the *df* selection in our models (S5 Table and S6 Table). In addition, the results of LOOCV also showed that the performance of model (2) was better than model (1) (S7 Table).

## Discussion

DF has become an increasingly important public health concern in Guangzhou, China in recent years, and in 2014 the number of DF cases represented the highest peak in the past 25 years [4]. A recent study suggested that urbanization, climate change, international trade and population movement were important factors that influenced this re-emergence of dengue in Guangzhou [5]. In order to improve early and rapid response to dengue outbreaks in Guangzhou, we combined dengue internet-based data (DBSI) with imported cases, temperature and rainfall to develop an early warning model. We found that inclusion of DBSI can improve the prediction of the base model reliant on traditional disease surveillance data. The results provide a new approach to developing a dengue early warning system in Guangzhou.

Many previous studies reported that climatic factors influenced DF transmission by directly or indirectly affecting each stage in the life cycle of the mosquito and the disease transmission [42,43]. In this study, we found that DF was positively correlated with average weekly minimum temperature at a lag of 9 weeks. This finding is generally in agreement with previous studies that indicate the crucial role of temperature in dengue transmission [44,45]. Possible reasons for this association with temperature are that higher temperature can reduce both mosquito maturity and reproduction time in favor of producing more mosquitoes in a shorter time [46]. We also found that rainfall has a nonlinear relationship with DF with a threshold of 149mm. This is consistent with several other studies that found that rainfall influenced vector abundance in subsequent weeks by creating more breeding habitats for mosquitoes [47]. On the other hand, it is also likely that heavy rain can destroy existing mosquito breeding sites and affect the maturation of mosquito eggs or larvae [48]. DF is not regarded as endemic in Guangzhou, and previous outbreaks were caused by imported cases [14]. Our study indicated that imported DF cases in the previous 5 weeks had a large impact on the local DF case numbers. The time delay could be the period of the life cycle of dengue transmission.

To the best of our knowledge, this study is the first one to investigate the relationship between DBSI and DF cases in China. We found that DBSI in the previous week had a positive linear relationship with reported DF cases, implying that internet-based search behavior may be a useful predictor of DF incidence. This is consistent with previous studies that investigated the relationship between Google Dengue Trends (GDT) and DF cases [23,24,49]. In one study in Singapore and Bangkok, Althouse et al. demonstrated that the internet search terms could successfully predict incidence and periods of large incidence of dengue with high accuracy. Their model using Google search data had an *r*^2^ = 0.948 and 0.943 for Singapore and Bangkok [23]. Chan et al. also observed in five countries that the models built on the fraction of Google search volume for dengue-related queries were able to adequately estimate true dengue activity, and the correlation between values predicted by models and the surveillance data was generally quite high, ranging from 0.82 to 0.99 [24].

As we mentioned in the introduction, the GFT firstly provided us an excellent example in 2003–3007 to illustrate the contribution of internet search data on the prediction of infectious diseases [18]. However, the GFT failed to successfully predict the seasonal and pandemic influenza in the USA during the 2012/2013 season [25]. It has been debated that the internet-based query might misrepresent the epidemic curve in practice [25,29]. Some researchers analyzed the reasons for this failure in the GFT model suggesting that the internet-based query system can be used as a supplement to, but not a substitute for, the traditional data collection and analysis [17,50]. Moreover, Gluskinet al. also demonstrated in Mexico that the model using GDT data in combination with relevant covariates (maximum temperature, logged precipitation) can significantly improve dengue prediction [49]. In our study, similar result was also found that the model including DBSI variable had a better performance than model without it. Collectively, these results indicate that integrating internet-based dengue query data into traditional disease surveillance can improve dengue prediction, providing us with a new approach for establishing an almost real-time early warning system. In this big data era when internet-based data are easily available and collected in almost real-time [51], its use as a supplement to traditional disease surveillance provides important progress towards establishing reliable early warning models allowing for more efficient and rapid control of infectious diseases.

We validated our model by comparing the predictive results with the surveillance dengue data in the last 8 weeks of study periods, and the results show good performance of the model. However, it has been suggested that the results of models using internet search queries need to be further validated by more advanced studies to control the relevant covariates (such as media basis, socio-economic and demographic factors) [50].

Some limitations of our study should be mentioned. First, the guidelines of dengue diagnosis and treatment were different before and after October 11^th^, 2014 in China. For example, a dengue virus NS1 antigen test was added to the new version as an important criterion, which might lead to some bias to our results. However, the influences of changing diagnosis guidelines on our results are limited, because only dengue cases in the last one and half months in 2014 were diagnosed by the new guidelines. Second, the study developed the prediction model using only a 4-year period of time-series data based on weekly data, and could only be validated for an 8-week period. It is advisable to use long-term time series data in model fitting in the future. Third, this study does not examine other potential confounding factors that may be associated with dengue incidence, such as environmental, socio-economic and demographic factors [52]. In addition, it has been suggested that internet searching behavior is susceptible to the impact of media reports [23,53], and we did not implement any measures to control for this. Studies could be conducted in the future to investigate how users interact with internet search sources for providing valuable information on potential biases and suggest mechanisms for improving the robustness of surveillance systems based on internet search queries.

## Conclusions

The present study suggests that the Dengue Baidu Search Index provides useful data for early prediction of a dengue outbreak. Such improvements in prediction and hence early warning are very important for improving prevention and control of dengue epidemics in the future.

## Supporting information

S1 Table Search terms from Baidu in Chinese and English. (DOCX)

S2 Table Dengue related Baidu search terms that were finally selected. (DOCX)

S3 Table Cross-correlation coefficients for local DF cases in Guangzhou and four predicting variables. (DOCX)

S4 Table Effective degrees of freedom of the smooth function terms in Model (2). (DOCX)

S5 Table Sensitivity analyses on the effects of *df* on GCVs in model (1). (DOCX)

S6 Table Sensitivity analyses on the effects of *df* on GCVs in model (2). (DOCX)

S7 Table The ICCs of model (1) and model (2) validated by the LOOCV method. (DOCX)
