# Supplementary material for: Dengue Baidu Search Index data can improve the prediction of local dengue epidemic: A case study in Guangzhou, China
Source: PLoS Negl Trop Dis. 2017 Mar 6;11(3):e0005354. doi: 10.1371/journal.pntd.0005354 (PMC5354435; doi:10.1371/journal.pntd.0005354)
Supplement: S1 Table — (DOCX) [file pntd.0005354.s001.docx]

Table S1. Search terms from Baidu in Chinese and English

| Terms | Terms |
| --- | --- |
| 被蚊子咬了怎么止痒  (how to relieve itching was bitten from a bitten by mosquito bite) | 关节疼痛  (arthralgia) |
| 持续高烧不退  (high fever) | 后背疼痛是什么原因  (what causes back pain) |
| 登革热  (dengue) | 花斑蚊  (aedes) |
| 登革热病  (dengue fever) | 花蚊子  (*aedes*) |
| 登革热病毒  (dengue virus) | 肌肉骨骼痛  (musculoskeletal pain) |
| 登革热病例  (dengue cases) | 肌肉痛  (courbature) |
| 登革热病是由哪种动物传染的  (which animal transmit dengue fever) | 皮疹  (erythra) |
| 登革热传染途径  (dengue transmission) | 驱蚊子  (mosquito repellent) |
| 登革热会传染吗  (will dengue fever infect others) | 蚊虫叮咬  (mosquito bites) |
| 登革热及登革出血热  (dengue and hemorrhagic fever) | 蚊虫叮咬用什么药  what to do for mosquito and insect bites |
| 登革热是什么  (what is dengue fever) | 蚊香  (mosquito coils) |
| 发热的原因  (cause of fever) | 蚊子  (mosquito) |
| 发烧  (fever) | 伊蚊  (aedes) |
