# Supplementary material for: Dengue Baidu Search Index data can improve the prediction of local dengue epidemic: A case study in Guangzhou, China
Source: PLoS Negl Trop Dis. 2017 Mar 6;11(3):e0005354. doi: 10.1371/journal.pntd.0005354 (PMC5354435; doi:10.1371/journal.pntd.0005354)
Supplement: S2 Table — (DOCX) [file pntd.0005354.s002.docx]

Table S2. Dengue related Baidu search terms that were finally selected

| Selected teams | Selected terms |
| --- | --- |
| 登革热会传染吗  (will dengue fever infect others) | 登革热病  (dengue fever) |
| 登革热  (dengue fever) | 伊蚊  (aedes) |
| 登革热病毒  (dengue virus) | 后背疼痛是什么原因  (what causes back pain) |
| 登革热传染途径  (dengue transmission) | 皮疹  (erythra) |
| 登革热是什么  (what is dengue fever) | 花斑蚊  (aedes) |
| 登革热病例  (dengue cases) |  |
