# Supplementary material for: Dengue Baidu Search Index data can improve the prediction of local dengue epidemic: A case study in Guangzhou, China
Source: PLoS Negl Trop Dis. 2017 Mar 6;11(3):e0005354. doi: 10.1371/journal.pntd.0005354 (PMC5354435; doi:10.1371/journal.pntd.0005354)
Supplement: S3 Table — (DOCX) [file pntd.0005354.s003.docx]

| Table S3. Cross-correlation coefficients for local DF cases in Guangzhou and four predicting variables. | | | | | | | | | |
| --- | --- | --- | --- | --- | --- | --- | --- | --- | --- |
| Variables | Lags | Coefficient | *P* |  |  | Variables | Lags | Coefficient | *P* |
| Minimum temperature | 1 | 0.41 | <0.001 |  |  | DBSI | 1 | 0.68 | <0.001 |
|  | 2 | 0.48 | <0.001 |  |  |  | 2 | 0.67 | <0.001 |
|  | 3 | 0.55 | <0.001 |  |  |  | 3 | 0.65 | <0.001 |
|  | 4 | 0.60 | <0.001 |  |  |  | 4 | 0.64 | <0.001 |
|  | 5 | 0.65 | <0.001 |  |  |  | 5 | 0.63 | <0.001 |
|  | 6 | 0.68 | <0.001 |  |  |  | 6 | 0.61 | <0.001 |
|  | 7 | 0.72 | <0.001 |  |  |  | 7 | 0.59 | <0.001 |
|  | 8 | 0.74 | <0.001 |  |  |  | 8 | 0.58 | <0.001 |
|  | 9 | 0.77 | <0.001 |  |  |  | 9 | 0.57 | <0.001 |
|  | 10 | 0.76 | <0.001 |  |  |  | 10 | 0.56 | <0.001 |
|  | 11 | 0.76 | <0.001 |  |  |  | 11 | 0.54 | <0.001 |
|  | 12 | 0.73 | <0.001 |  |  |  | 12 | 0.52 | <0.001 |
|  | 13 | 0.69 | <0.001 |  |  |  | 13 | 0.50 | <0.001 |
|  | 14 | 0.65 | <0.001 |  |  |  | 14 | 0.48 | <0.001 |
|  | 15 | 0.60 | <0.001 |  |  |  | 15 | 0.46 | <0.001 |
|  | 16 | 0.54 | <0.001 |  |  |  | 16 | 0.43 | <0.001 |
| Cumulative rainfall | 1 | 0.01 | 0.923 |  |  | Imported DF cases | 1 | 0.43 | <0.001 |
|  | 2 | 0.03 | 0.702 |  |  |  | 2 | 0.44 | <0.001 |
|  | 3 | 0.08 | 0.241 |  |  |  | 3 | 0.43 | <0.001 |
|  | 4 | 0.13 | 0.062 |  |  |  | 4 | 0.43 | <0.001 |
|  | 5 | 0.19 | 0.010 |  |  |  | 5 | 0.45 | <0.001 |
|  | 6 | 0.24 | <0.001 |  |  |  | 6 | 0.44 | <0.001 |
|  | 7 | 0.28 | <0.001 |  |  |  | 7 | 0.41 | <0.001 |
|  | 8 | 0.34 | <0.001 |  |  |  | 8 | 0.39 | <0.001 |
|  | 9 | 0.39 | <0.001 |  |  |  | 9 | 0.38 | <0.001 |
|  | 10 | 0.46 | <0.001 |  |  |  | 10 | 0.34 | <0.001 |
|  | 11 | 0.51 | <0.001 |  |  |  | 11 | 0.29 | <0.001 |
|  | 12 | 0.53 | <0.001 |  |  |  | 12 | 0.25 | <0.001 |
|  | 13 | 0.52 | <0.001 |  |  |  | 13 | 0.24 | <0.001 |
|  | 14 | 0.52 | <0.001 |  |  |  | 14 | 0.18 | 0.012 |
|  | 15 | 0.51 | <0.001 |  |  |  | 15 | 0.15 | 0.044 |
|  | 16 | 0.51 | <0.001 |  |  |  | 16 | 0.08 | 0.291 |
