# Supplementary material for: Dengue Baidu Search Index data can improve the prediction of local dengue epidemic: A case study in Guangzhou, China
Source: PLoS Negl Trop Dis. 2017 Mar 6;11(3):e0005354. doi: 10.1371/journal.pntd.0005354 (PMC5354435; doi:10.1371/journal.pntd.0005354)
Supplement: S4 Table — (DOCX) [file pntd.0005354.s004.docx]

Table S4. Effective degrees of freedom of the smooth function terms in Model (2)

| Smooth terms | edf | *F* |
| --- | --- | --- |
| s(Minimum temperature averaged over previous 9 weeks) | 1.84 | 5.54* |
| s(Cumulative rainfall over previous 12 weeks) | 2.69 | 7.28* |
| s(BSI over previous week) | 1.00 | 33.01* |
| s(Imported DF over previous 5 weeks) | 1.93 | 7.55* |
| s(Local DF over previous week) | 1.98 | 73.01* |
| s(week) | 3.24 | 45.61* |

edf : Effective degree of freedom(edf>1 indicates a non-linear relationships); *F* value is an approximate *F*-test; *:*p*-value less than 0.05.
