# Supplementary material for: Dengue Baidu Search Index data can improve the prediction of local dengue epidemic: A case study in Guangzhou, China
Source: PLoS Negl Trop Dis. 2017 Mar 6;11(3):e0005354. doi: 10.1371/journal.pntd.0005354 (PMC5354435; doi:10.1371/journal.pntd.0005354)
Supplement: S5 Table — (DOCX) [file pntd.0005354.s005.docx]

Table S5. Sensitivity analyses on the effects of *df* on GCVs in model (1)

| *df*s of variables in model (1) | | *df*s of variables in model (1) | | | | | | | | | | |
| --- | --- | --- | --- | --- | --- | --- | --- | --- | --- | --- | --- | --- |
|  |  | 2 ^a^ | | |  | 3 ^a^ | | |  | 4 ^a^ | | |
|  |  | 2^b^ | 3^b^ | 4^b^ |  | 2^b^ | 3^b^ | 4^b^ |  | 2^b^ | 3^b^ | 4^b^ |
| 2^c^ | 2^d^ | 20.07 | 19.39 | 21.76 |  | 20.36 | 20.19 | 19.02 |  | 19.55 | 20.50 | 19.35 |
|  | 3 ^d^ | 19.34 | 19.28 | 19.91 |  | 20.44 | 20.52 | 19.59 |  | 19.21 | 19.30 | 19.93 |
|  | 4 ^d^ | 18.78 | 18.94 | 20.38 |  | 18.95 | 18.73 | 19.12 |  | 19.46 | 19.00 | 20.15 |
| 3 ^c^ | 2 ^d^ | 19.13 | 19.24 | 20.09 |  | 19.35 | 19.00 | 19.91 |  | 21.12 | 21.39 | 20.27 |
|  | 3 ^d^ | 19.34 | 19.22 | 20.86 |  | 18.63 | 18.41 | 19.78 |  | 18.81 | 18.99 | 19.37 |
|  | 4 ^d^ | 19.89 | 21.17 | 21.03 |  | 20.31 | 18.85 | 19.99 |  | 20.94 | 21.68 | 20.04 |
| 4 ^c^ | 2 ^d^ | 20.52 | 19.31 | 21.62 |  | 19.65 | 18.97 | 19.49 |  | 21.21 | 20.67 | 19.47 |
|  | 3 ^d^ | 20.31 | 20.27 | 19.58 |  | 19.04 | 20.03 | 19.36 |  | 19.40 | 19.74 | 20.06 |
|  | 4 ^d^ | 20.40 | 20.39 | 20.94 |  | 21.77 | 20.52 | 20.84 |  | 21.49 | 21.80 | 20.81 |

a: *df* of minimum temperature; b: *df* of cumulative rainfall; c: *df* of local DF; d: *df* of import DF; GCV: Generalized Cross Validation score
