# Supplementary material for: Dengue Baidu Search Index data can improve the prediction of local dengue epidemic: A case study in Guangzhou, China
Source: PLoS Negl Trop Dis. 2017 Mar 6;11(3):e0005354. doi: 10.1371/journal.pntd.0005354 (PMC5354435; doi:10.1371/journal.pntd.0005354)
Supplement: S6 Table — (DOCX) [file pntd.0005354.s006.docx]

Table S6. Sensitivity analyses on the effects of *df* on GCVs in model (2)

| *df* of DBSI in model (2) | GCV |
| --- | --- |
| 2 | 8.09 |
| 3 | 7.62 |
| 4 | 7.85 |

Note: The dfs of all other variables in model (2) were set to 3.

GCV: Generalized Cross Validation score.
