# Supplementary material for: Dengue Baidu Search Index data can improve the prediction of local dengue epidemic: A case study in Guangzhou, China
Source: PLoS Negl Trop Dis. 2017 Mar 6;11(3):e0005354. doi: 10.1371/journal.pntd.0005354 (PMC5354435; doi:10.1371/journal.pntd.0005354)
Supplement: S7 Table — (DOCX) [file pntd.0005354.s007.docx]

Table S7. The ICCs of model (1) and model (2) validated by the LOOCV method

| model | ICC | *P* |
| --- | --- | --- |
| Model (1) | 0.79 | <0.001 |
| Model (2) | 0.86 | <0.001 |

ICC: Intraclass Correlation Coefficient
